# Preparing healthcare education for an AI-augmented future

**DOI:** 10.1038/s44401-024-00006-z

**Published:** 2024-12-23

**Authors:** Jiajie Zhang, Susan H. Fenton

**Affiliations:** University of Texas Health Science Center at Houston, Houston, TX USA

**Keywords:** Health care, Education

## Abstract

Artificial intelligence (AI) fundamentally transforms healthcare education as a knowledge enterprise, creating a distributed cognitive system composed of the human brain, which remains relatively unchanged, and AI-based knowledge and cognitive functions, which have accelerated exponentially in scale and power. Education must focus on developing skills to collaborate with AI and on achieving outcomes like problems solved and discoveries made. Curriculum and education policies also need to adapt to this transformation.

## Education as a knowledge enterprise

Education, in general, is fundamentally a knowledge enterprise that revolves around creating, disseminating, and applying knowledge. It serves as the vehicle through which societies transmit accumulated wisdom, skills, and values to new generations, fostering intellectual growth and preparing individuals for societal participation. In this activity, teaching and learning are deeply rooted in cognitive processes—engaging memory, attention, perception, language, reasoning, decision-making, and critical thinking. Effective teaching requires structuring information that aligns with how the brain naturally processes, stores, and retrieves knowledge. On the other hand, learning involves the active construction of meaning, where students integrate new information with prior understanding, making learning an adaptive and iterative process. In the age of artificial intelligence (AI), the cognitive processes underlying learning become even more crucial. With AI capable of automating a wide variety of knowledge-based tasks, the role of healthcare education must shift toward cultivating higher-order thinking skills—such as meta-cognition, creativity, contextual and situated thinking, and ethical reasoning—that machines cannot easily replicate. This makes education not only about knowledge transfer but also about enhancing human cognitive capabilities to navigate and shape a rapidly evolving technological landscape.

## The cognitive revolution is fundamentally changing the nature of knowledge and cognitive process

We are at the beginning of the third fundamental economic transformation in human history—the *Cognitive Revolution*, triggered by recent AI breakthroughs and driven by massive data, universal connectivity, and powerful computing. The Cognitive Revolution is liberating people from cognitive labor. It is as fundamental as the two previous economic transformations: the Agricultural Revolution and the Industrial Revolution. The Agricultural Revolution, biological in nature, took place around 10,000 BC, liberating people from food insecurity by farming crops and animals. It marked the shift from hunting and food gathering to settled agricultural communities and sustained food supply, fundamentally changing human life. The Industrial Revolution, physical in nature, began about 200 years ago, releasing people from grueling physical labor through machines, transforming economies by enabling mass production and mechanization. Similarly, today’s Cognitive Revolution is transforming economies and industries by enabling AI to perform cognitive tasks traditionally handled by humans.

**Fig. 1:**
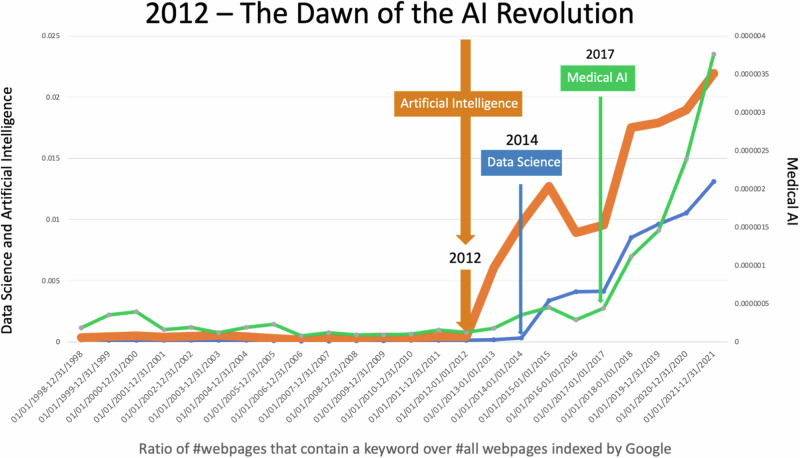
The proportion of webpages that contain a keyword (including synonyms) in the Google universe.

The AI Revolution in 2012 (Fig. 1) was the seed for the broader Cognitive Revolution when the deep learning model AlexNet developed by^1^ achieved landmark success in the ImageNet competition^2^. This deep learning model dramatically outperformed competing models, demonstrating the power of deep neural networks in processing visual data. This success sparked a massive wave of advancements in AI, leading to innovations in speech recognition, natural language processing, autonomous driving, and medical diagnostics, among many others.

AI systems can now learn, analyze, and make decisions, automating a wide range of cognitive labor and reshaping industries. In medicine, AI has revolutionized diagnostic procedures, enabling early detection of diseases through image analysis and predictive analytics. AI-powered tools assist in developing personalized treatment plans by analyzing patient data and predicting individual responses to therapies. Nursing benefits from AI through enhanced patient monitoring systems that use predictive analytics to prevent adverse events. AI also optimizes resource allocation, streamlines patient flow, and improves operational efficiencies in healthcare administration, such as ambient systems that convert patient-clinician conversations into structured clinical notes and suggest diagnoses and treatment plans. AI’s influence is not limited to automating certain cognitive tasks; it is driving innovation across medicine, nursing, dentistry, and numerous other fields, fundamentally transforming the way we learn, work, and live.

At the heart of this revolution is AI’s ability to do cognitive tasks that were traditionally performed by humans only—such as learning, memory, language, reasoning, decision-making, and problem-solving—on a vast scale. AI is not only a tool for advancing scientific discovery but as a fundamental force reshaping industries and the global economy. AI’s unprecedented ability to perform complex cognitive functions and generate insights from massive datasets positions it as a critical engine for future economic growth.

## The nature of education needs to be redefined

AI is revolutionizing education by fundamentally transforming it as a cognitive process. Initially a subfield within computer science, AI now permeates every educational field, reshaping how we approach learning and teaching. With AI handling routine cognitive tasks, education systems must evolve beyond traditional methods focused on rote memorization and information retention. Education should focus on developing higher-order cognitive skills that AI cannot easily replicate, such as metacognitive strategies where students learn to evaluate and regulate their own thinking processes.

AI is also revolutionizing education as a knowledge enterprise. One prominent example is the development of large language models (LLMs) trained on nearly all public knowledge created in human civilization. These models can perform highly advanced memory tasks, retrieving and synthesizing information from vast datasets with remarkable accuracy and speed. Beyond mere data recall, LLMs can generate new insights, facilitate complex problem-solving, and support interdisciplinary learning by drawing connections across diverse fields of knowledge. By leveraging the capabilities of LLMs, educators can create more interconnected and comprehensive learning environments that encourage students to synthesize information across disciplines. This approach transforms education into a dynamic, lifelong pursuit of knowledge and skill development, preparing learners for a future where human-AI collaboration is the norm.

## Human intelligence as distributed cognition

Human cognition rarely functions as an isolated, encapsulated brain without interacting with the outside world and with other people. Human cognition is a distributed cognitive system with knowledge and processes distributed between human biological brains and the external world, across groups of individuals, and across space and time^3–5^. The external knowledge and structures of distributed cognition are cognitive artifacts: sophisticated tools humans create to augment human cognitive processes, enhancing rather than replacing our intellectual capabilities^6^. These artifacts have been integral to human development, from the early use of stones for counting to the advanced computing devices of today. Each iteration has served as an extension of our cognitive faculties, facilitating tasks that would be arduous or impossible to perform unaided. For instance, the abacus allowed for complex calculations long before the advent of modern calculators, demonstrating an early form of cognitive augmentation. Similarly, the development of written language revolutionized communication, enabling the recording and dissemination of knowledge across generations. Mathematics provided a foundational framework for logical reasoning and scientific discovery, underpinning technological advancements that continue to shape our world.

**Fig. 2:**
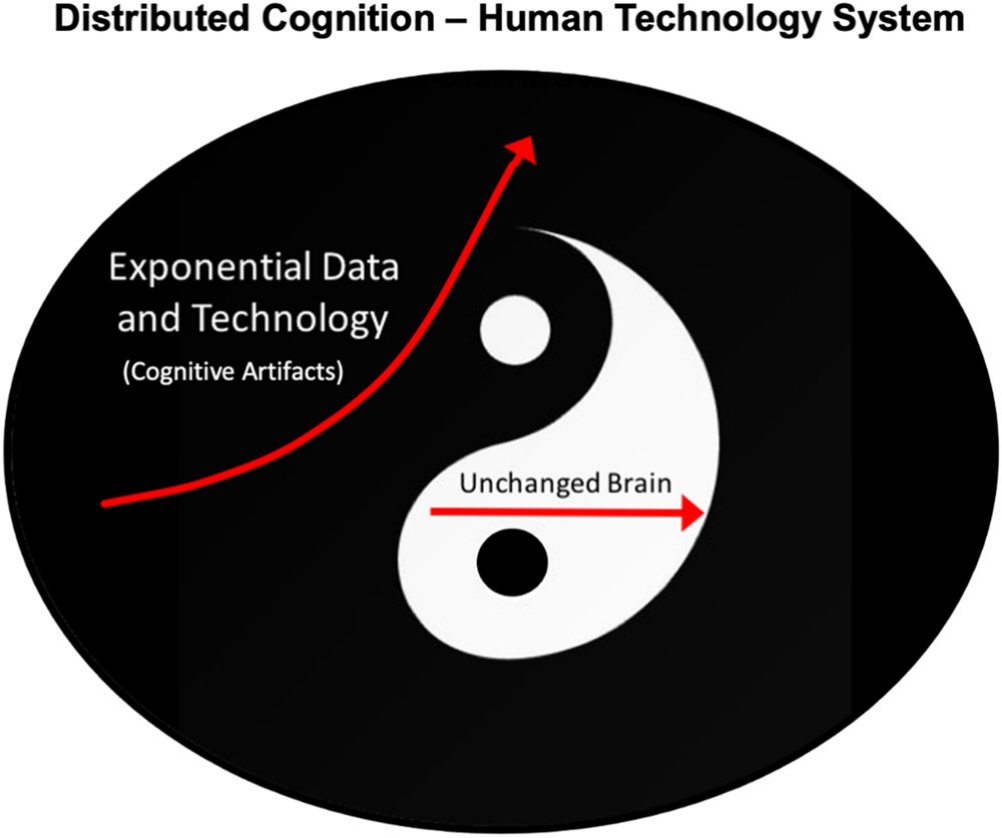
Distributed Cognition system composed of the human brain and technology. The cognitive capacities of the human biological brain have remained basically unchanged over the millennia. In contrast, data and technology outside the human brain have accelerated exponentially over the past few decades.

The framework of distributed cognition (Fig. 2) shows the dynamic interplay between rapidly advancing cognitive artifacts and the relatively stable nature of the human biological brain. The human biological brain’s cognitive capacities have remained unchanged over the millennia. In contrast, data and technology outside the human brain have accelerated exponentially over the past few decades^7^. Although the biological brain remains relatively static, the Distributed Cognition system composed of the brain and technology grows exponentially in capacity and ability. Additionally, while technology is growing at an unprecedented rate, this does not equate to surpassing human cognitive abilities but rather an augmentation of them. Cognitive artifacts amplify human intelligence by converging technology’s external capabilities with the brain’s internal processing power. Tools like LLMs are not independent entities but are deeply integrated with and dependent on human guidance and input. AI, as embodied by LLMs and other cognitive artifacts, is not on a trajectory to overtake human intelligence but to collaborate with and enhance it within a distributed cognition system.

## From interdisciplinary to AI-augmented education: the impact of AI

In the Cognitive Age, the traditional boundaries between disciplines have been dissolving, and even the interdisciplinary approaches that once connected and overlapped various fields have transformed. The exponentially grown, ever-increasingly capable technology side of distributed cognition is a unified knowledge base encompassing all human disciplines in a single, integrated system. AI’s comprehensive knowledge base spans natural sciences, social sciences, engineering, humanities, and the arts, providing unprecedented resources and tools for scientific research and creative endeavors. Although the human side of distributed cognition may become specialized in newly emerging areas, the technology side that integrates and unifies all disciplines will drive education, learning, and innovation in the foreseeable future.

The 2024 Nobel Prizes in Physics and Chemistry^8^ given to AI researchers highlight a fundamental shift, recognizing AI as a vital partner in advancing scientific knowledge, not just a tool. AI’s capabilities extend far beyond data processing; it facilitates complex problem-solving, model simulations, and the generation of novel insights across disciplines. For instance, in physics and chemistry, AI-driven models are unlocking new areas of understanding by simulating molecular behaviors and predicting material properties with unparalleled precision.

The implications of AI in education are equally transformative. Traditional educational models, often compartmentalized by disciplines or loosely overlapped interdisciplines, are evolving into integrated frameworks reflecting AI-augmented knowledge’s interconnected nature in the Cognitive Age. This AI-augmented approach emphasizes the synthesis of information across diverse fields. It allows learners to use the unified knowledge base created by AI and encourages them to draw connections and apply their understanding in multifaceted contexts in the distributed cognition system composed of humans and AI.

AI empowers educators to create dynamic, personalized learning environments that adapt to the needs of individual students. Through advanced data analytics and machine learning algorithms, educators can tailor instructional methods, provide real-time feedback, and foster an inclusive learning experience that promotes critical thinking, creativity, and problem-solving skills.

Moreover, AI-augmented education prepares learners for a future where human-AI collaboration is the norm. As AI continues to augment human intelligence, developing emotional intelligence, social cognition, and ethical reasoning becomes increasingly important. Education systems must, therefore, focus on cultivating these higher-order cognitive skills, ensuring that learners are equipped to navigate the complexities of an AI-augmented world.

The transition from disciplinary and interdisciplinary to a unified, AI-augmented education reflects a broader paradigm shift in understanding and engaging with knowledge. By embracing the Cognitive Revolution, educational institutions can foster a culture of lifelong learning, where disciplinary boundaries do not confine the pursuit of knowledge but are a continuous journey of discovery and innovation in the universe of distributed cognition composed of humans and AI, with the AI component constituting a unified knowledge base for all disciplines.

## A Case Study: Transcending interdisciplinary to AI-augmented education

### Biomedical informatics as an interdisciplinary field

*Biomedical Informatics* studies the acquisition, storage, communication, processing, integration, analysis, mining, retrieval, interpretation, and presentation of data and determines how to transform data (meaningless symbols) to information (interpreted data), to knowledge (validated information), and to intelligence (actionable knowledge), with the aim of solving problems in disease prevention, healthcare delivery, and biomedical discovery.

At the McWilliams School of Biomedical Informatics (MSBMI) at the University of Texas Health Science Center at Houston (UTHealth Houston), interdisciplinary education has been a cornerstone of its mission. Biomedical informatics at MSBMI covers the entire spectrum of biological scales—from small molecules, genes, proteins, and cells, to tissues and organs, and to individuals and populations. Its faculty are from clinical practice (medicine, nursing, dentistry, and pharmacy), the basic biomedical sciences, public and population health, computer science and engineering, mathematics and biostatistics, cognitive science, the social and behavioral sciences, healthcare management, and law. Students at MSBMI come from similarly varied backgrounds. Some are healthcare professionals looking to deepen their data science and informatics expertise, whereas others are engineers or computer scientists aiming to apply their technical skills to the health domain.

The courses at MSBMI reflect this interdisciplinary approach, with over sixty courses covering bioinformatics, AI, data science, imaging, clinical informatics, management, and social sciences, ensuring that students understand how different fields contribute to healthcare innovation. Moreover, the school offers a variety of degree programs—ranging from graduate certificates to master’s and doctoral degrees—that cater to a broad range of student needs and backgrounds, further enhancing the interdisciplinary learning environment.

Faculty and students work on projects that span various domains, such as using deep learning to predict clinical outcomes like hospital readmissions or stroke onset, deploying statistical methods to uncover the genomic basis of diseases, and integrating clinical data with biological and imaging data to improve patient care. These projects require expertise from medicine, engineering, data science, and beyond, making interdisciplinary collaboration a necessity rather than a choice.

In this complex and ever-evolving landscape, interdisciplinary education at MSBMI prepares students to become leaders in the field, capable of integrating knowledge from diverse areas to innovate and drive the future of healthcare.

### Transcending interdisciplinary to AI-augmented education

Now, MSBMI is transcending its traditional interdisciplinary approach by embracing AI-augmented education. This new paradigm goes beyond merely combining disciplines by integrating AI into every facet of the learning experience. This change recognizes the need for students to develop a comprehensive understanding of how AI serves as an integrated and unified knowledge base encompassing all disciplines. Students are encouraged to learn how to utilize this extensive knowledge base and contribute new insights and discoveries to it. Additionally, AI’s role as a cognitive process that performs various high-level cognitive tasks is emphasized, with students being taught how to leverage these AI functions for their learning and research endeavors. This AI-augmented approach to education ensures that learners are adept at navigating and harnessing the power of AI both as a vast repository of knowledge and as a sophisticated cognitive tool.

AI is being embedded into the curriculum at MSBMI to provide students with hands-on experience leveraging machine learning, natural language processing, and data analytics to solve complex healthcare challenges. This integration ensures that learners are proficient in applying AI technologies to real-world scenarios, enhancing their ability to innovate and drive advancements in patient care, disease prevention, and biomedical discovery. Courses are designed to foster a deep understanding of AI’s potential and limitations, encouraging students to think critically about ethical implications and the societal impact of AI in healthcare. By emphasizing the importance of emotional intelligence, social cognition, and ethical reasoning, MSBMI ensures its graduates are technically adept and prepared to navigate an AI-augmented world’s moral and social complexities.

Considering these advancements, MSBMI is also revising its policies on using AI in coursework, class projects, master’s theses, and doctoral dissertations. This revision emphasizes three key areas: first, the cultivation of essential learning skills and foundational knowledge necessary for students to effectively and critically think and navigate within the AI knowledge base; second, a strong focus on the tangible outcomes of learning, such as the problems solved and the discoveries made; and third, an adherence to accreditation requirements to ensure that all educational standards are met. By aligning AI integration with these principles, the school ensures that students not only become proficient in AI technologies but also achieve meaningful academic and professional milestones.

## Conclusions

AI-augmented healthcare education goes beyond the mere transmission of knowledge. AI is generating an unprecedented transformation, unifying and expanding the knowledge base to dimensions previously unattainable by human capacity alone. AI is capable of performing a wide variety of high-level cognitive functions traditionally exclusive to human intellect, thereby transforming both the repository of knowledge and the very processes of cognition.

This paradigm shift mandates a reformation of healthcare educational curricula. Embedding AI at the nucleus of the learning experience is imperative, furnishing students with direct experience in AI. Such integration is essential to ensure learners are proficient in deploying AI technologies to address real-world challenges, thereby augmenting their capacity for innovation and driving advancements across various disciplines, not just in healthcare but also in engineering, natural sciences, social sciences, and humanities.

Furthermore, there is an urgent need to revise healthcare educational programs to reflect this new reality. These programs must prioritize cultivating critical learning skills and problem-solving competencies over mere content acquisition. Emphasis should be placed on the tangible outcomes of the educational process, such as problems solved and discoveries made. Such a focus will better equip students to navigate the complexities of an AI-augmented world, ensuring they are prepared to utilize AI’s potential while considering its ethical and societal ramifications.

The future of healthcare education lies in its adaptive capacity to integrate AI as a distributed cognition system where human intelligence and artificial intelligence synergistically contribute to knowledge creation and problem-solving.

## Data Availability

No datasets were generated or analysed during the current study.
